# Pharmacokinetics of Tyrosol Metabolites in Rats

**DOI:** 10.3390/molecules21010128

**Published:** 2016-01-21

**Authors:** Da-Hye Lee, Yang-Ji Kim, Min Jung Kim, Jiyun Ahn, Tae-Youl Ha, Sang Hee Lee, Young Jin Jang, Chang Hwa Jung

**Affiliations:** 1 Research Group of Metabolic Mechanism, Korea Food Research Institute, Seongnam 463-746, Korea; dlekgp26@nate.com (D.-H.L.); pretty37373@naver.com (Y.-J.K.); kmj@kfri.re.kr (M.J.K.); jyan@kfri.re.kr (J.A.); tyhap@kfri.re.kr (T.-Y.H.); shlee@kfri.re.kr (S.H.L.); jyj616@kfri.re.kr (Y.J.J.); 2 Department of Food Biotechnology, Korea University of Science and Technology, Seongnam 463-746, Korea

**Keywords:** tyrosol, pharmacokinetics, metabolites, tyrosol-4-sulfate

## Abstract

Tyrosol is considered a potential antioxidant; however, little is known regarding the pharmacokinetics of its metabolites. To study the pharmacokinetics of tyrosol-derived metabolites after oral administration of a single dose of tyrosol, we attempted to identify tyrosol metabolites in rat plasma by using ultra-performance liquid chromatography and quadrupole time-of-flight mass spectrometry (UPLC-Q-TOF-MS). Two tyrosol metabolites (M1 and M2) were detected in the plasma. M1 was identified as tyrosol-4-sulfate (T4S) with an [M − H]^−^ ion at *m*/*z* 217. While M2 showed an [M − H]^−^ ion at *m*/*z* 151.0, its metabolite was not identified. Pharmacokinetic analysis of T4S and M2 showed rapid uptake after oral administration of tyrosol within 1 h. The metabolites were rapidly distributed in most organs and tissues and eliminated within 4 h. The greatest T4S deposition by tissue weight was observed in the liver, followed by the kidney and spleen, while M2 was most concentrated in the kidney followed by the liver and spleen. These findings indicate that T4S and M2 were distributed mainly in tissues with an abundant blood supply and were rapidly excreted in urine.

## 1. Introduction

Phytochemicals are a large group of plant-derived compounds that may be beneficial in the prevention of various disorders such as inflammation, obesity, cancer, and neurodegenerative and cardiovascular diseases [1,2,3,4]. Owing to their potentially beneficial impact on human health, phytochemicals have gained increasing attention. Although their beneficial effects are dependent on their uptake and distribution in the body, the pharmacokinetics of most phytochemicals has not been elucidated or studied extensively.

Tyrosol is a phenylethanoid and phenolic antioxidant present in a variety of natural sources [5,6]. The principal sources in the human diet are olive oil and wine [7,8]. It is also one of the natural phenols in the traditional Korean rice wine makgeolli and is present in a greater proportion than other phenolic compounds [9]. Tyrosol has various biological activities. Recently, tyrosol was able to increase the longevity of *C. elegans* [10] and could ameliorate hyperglycemia in diabetic rats [11]. Previously, our data showed that tyrosol prevents endoplasmic reticulum (ER) stress-induced apoptosis in pancreatic β-cells through JNK signaling, suggesting that tyrosol could be a potential therapeutic candidate for the amelioration of type 2 diabetes [12].

Tyrosol is a potential antioxidant; however, not much is known regarding its metabolism and pharmacokinetics [13,14]. It is poorly metabolized to glucuronide conjugates in human hepatoma HepG2 cells, with no detection of sulfated metabolites [14]. Previous pharmacokinetic studies on tyrosol focused on the bioavailability of phenolic compounds in olive oil after acid and enzyme hydrolysis [15,16,17]. However, the exact pharmacokinetics of tyrosol metabolites was not fully explored.

Since pharmacokinetics of phytochemicals is important in understanding their mechanism of action, the aim of this study was to identify the tyrosol metabolites and determine their pharmacokinetics. The distribution of tyrosol metabolites in various tissues was also investigated in rats following the oral administration of tyrosol.

## 2. Results and Discussion

### 2.1. Identification of Tyrosol Metabolites

**Figure 1:**
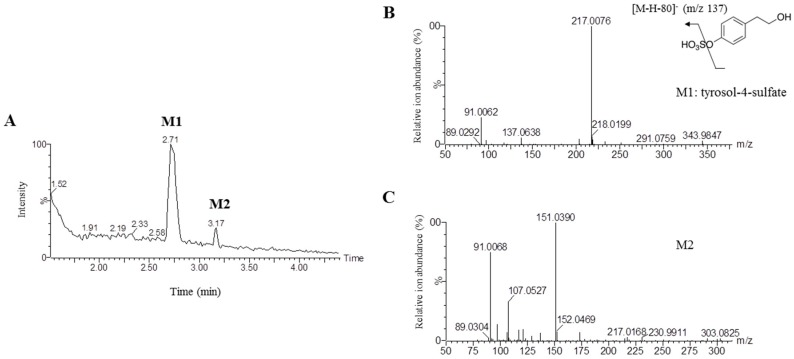
LC-MS/MS analysis of tyrosol metabolites in rat plasma following oral administration of tyrosol. Total ion chromatogram of tyrosol metabolites in rat plasma (**A**); Mass spectra of [M − H]^−^ ions and fragmentation schemes for M1 (**B**) and M2 (**C**). M1 and M2 are tyrosol metabolites. Plasma samples were collected at different time intervals post-dosing (0, 15, 20, 30, 60, 120, 180, and 240 min). To enable identification of tyrosol metabolites, pooled plasma samples were obtained at all time points.

**Figure A1:**
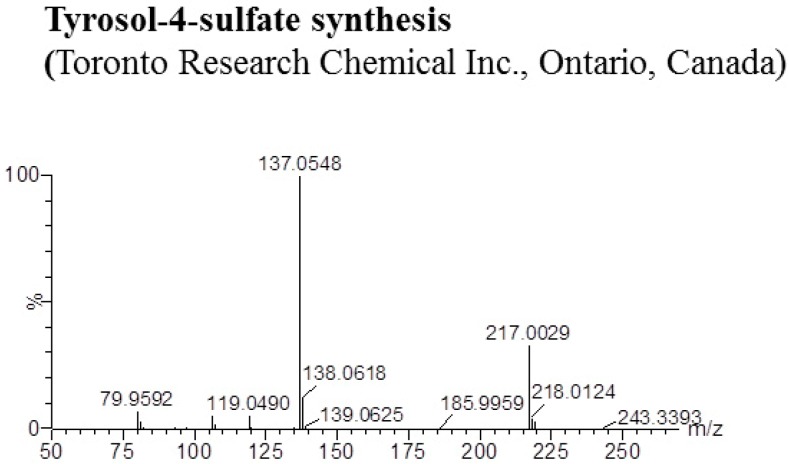
LC-MS anlaysis of synthesized tyrosol-4-sulfate.

Olive oil is one of the key dietary fats in the Mediterranean diet and is known to lower cardiovascular risk [18]. Tyrosol is a major component of olive oil and may contribute significantly to its touted health benefits. Recently, a study reported that tyrosol attenuated hepatic statuses in diet-induced obese mice by maintaining the redox potential and lowering reactive oxygen species (ROS) levels [19]. Despite its promising biological activity, mainly its antioxidant properties, little is known regarding the metabolism and pharmacokinetics of orally administered tyrosol. To investigate the pharmacokinetics of tyrosol metabolites in rat plasma, blood samples were collected via orbital venous plexus at various time intervals after a single-dose oral administration of tyrosol. In order to determine the potential metabolites, blood samples before and after administration of tyrosol were compared by UPLC-ESI/Q-TOF-MS. Two major metabolites (M1 and M2) were detected and characterized by the [M − H]^−^ ion at *m*/*z* 217.0 and the [M − H]^−^ ion at *m*/*z* 151.0, respectively (Figure 1A–C). Based on the MS/MS spectra, M1 was identified as tyrosol-4-sulfate (T4S). However, we could not identify the other possible tyrosol-derived metabolite of M2, and although we expected to detect other metabolites, such as tyrosol glucuoronides or methylated tyrosol, in the plasma, these metabolites were not detected in our analysis. To further study its pharmacokinetics, we synthesized additional T4S and demonstrated via LC-MS that its molecular weight and ion fragmentation patterns were consistent with that of M1 (Figure A1).

### 2.2. Pharmacokinetic Study

**Figure 2:**
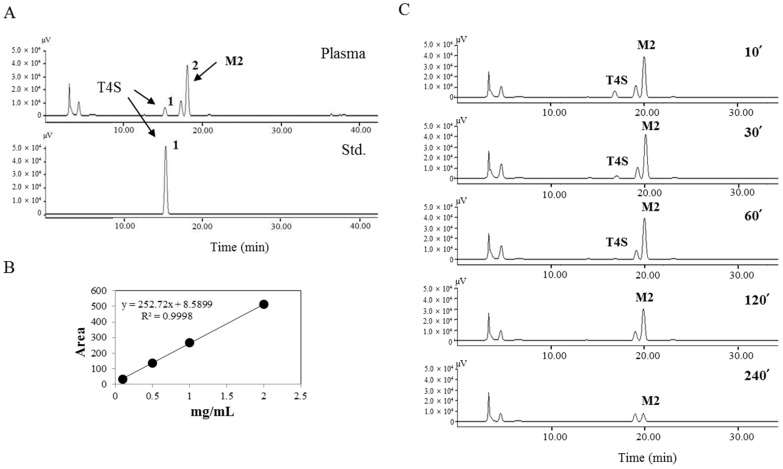
HPLC chromatogram of tyrosol metabolites in rat plasma. (**A**) Analysis of tyrosol metabolites in rat plasma following oral administration of tyrosol and standard of tyrosol-4-sulfate (T4S) using HPLC; (**B**) Standard curve of T4S; (**C**) HPLC chromatograms of tyrosol metabolites in plasma obtained at different time points following oral administration of tyrosol obtained by measuring absorbance at 280 nm.

To understand the time-dependent changes in the concentration of tyrosol metabolites, the level of metabolites in plasma samples was measured by HPLC at different time points (15, 30, 60, 120, and 240 min) after tyrosol administration. Tyrosol metabolites were found to elute without any interference from endogenous components in the plasma (Figure 2A). The correlation coefficient of the calibration curve was above 0.99 (Figure 2B), suggesting good linearity within the tyrosol-4-sulfate concentration range of 0.1–2 mg/mL. The inter- and intra-day precision and accuracy were determined by replicate analysis of the samples at two concentrations of each metabolite: 0.1 and 2 mg/mL tyrosol-4-sulfate. The stability of the metabolites was determined at room temperature over 24 h. The precision and accuracy were found to be well within the acceptance range of 10% and the metabolites in the plasma were found to be stable for 24 h at room temperature (data not shown). The HPLC chromatogram of plasma obtained at different time points after administration of tyrosol was shown in Figure 2C.

**Figure 3:**
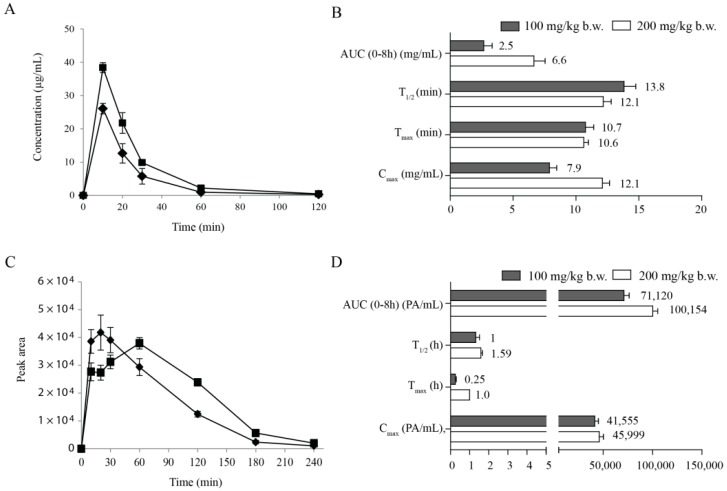
Pharmacokinetic analysis of tyrosol metabolites in rat plasma. (**A**) Plasma concentration–time profiles of tyrosol-4-sulfate; (**B**) Plasma pharmacokinetics profiles of tyrosol-4-sulfate; (**C**) Plasma concentration–time profiles of M2; (**D**) Plasma pharmacokinetic profiles of M2. Tyrosol was orally administrated to rats at a dose of 100 (♦) or 200 mg/kg body weight (■). PA: peak area; C_max_: maximum recorded concentration; T_max_: time to reach C_max_; AUC (area under the curve): a measure of drug exposure; T_1/2_: elimination half-life. Each point represents mean ± SD (*n* = 3).

The mean plasma concentration–time profiles showed that tyrosol metabolites were rapidly absorbed and their absorption decreased gradually at later time points (Figure 3A,C). The T_max_ values of T4S were 10.7 min and 10.6 min and the maximum plasma concentration (C_max_) values were 7.9 and 12.1 mg/mL at dosages of 100 and 200 mg per kg body weight, respectively (Figure 3B). In contrast, M2 was detectable even at 120 min and the C_max_ of M2 was dependent on the administered concentration (Figure 3C). Although we could not determine the exact concentration of M2 in plasma samples, the time to maximum plasma concentration (T_max_) of M2 was 0.25 h and 1.0 h and the terminal half-life (T_1/2_) was 1.0 h and 1.59 h at 100 and 200 mg/kg, respectively (Figure 3D). These results indicate that tyrosol metabolites T4S and M2 were formed shortly after oral administration in rats and might be absorbed directly through the stomach wall.

**Figure 4:**
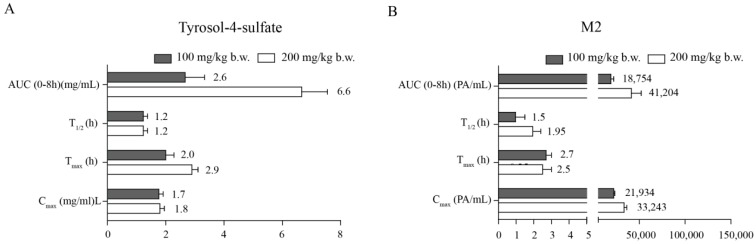
Pharmacokinetic analysis of tyrosol metabolites in rat urine. (**A**) Urine pharmacokinetics profiles of tyrosol-4-sulfate; (**B**) Urine pharmacokinetics profiles of M2. Tyrosol was orally administrated to rats at a dose of 100 (♦) or 200 mg/kg body weight (■). PA: peak area; C_max_: maximum recorded concentration; T_max_: time to reach C_max_; AUC (area under the curve): a measure of drug exposure; T_1/2_: elimination half-life. Each point represents mean ± SD (*n* = 3).

Urine samples were collected at 0–1, 1–2, 2–4, 4–8, and 8–24 h after administration of tyrosol. T4S and M2 were detected in urine by HPLC analysis (data not shown), suggesting that tyrosol is excreted in the form of T4S and M2. The T_max_ of T4S and M2 in urine demonstrated a similar pattern in the range of 2–2.9 h and 2–2.7 h, respectively, and were rapidly eliminated by 12 h (Figure 4A,B).

### 2.3. Tissue Distribution of Tyrosol-Derived Metabolites

**Figure 5:**
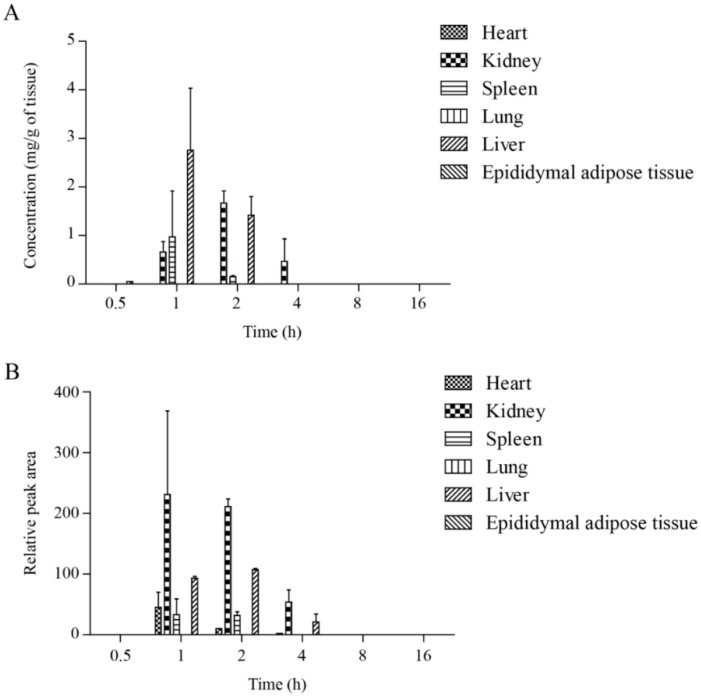
Tissue distribution of tyrosol-4-sulfate (**A**) and M2 (**B**). Tissue samples (heart, kidney, spleen, lung, liver, epididymal adipose tissue) were collected at different time intervals post-dosing (0, 0.5, 1, 2, 4, 8, and 16 h) and the concentration of tyrosol-4-sulfate and M2 was measured. Each point represents mean ± SD (*n* = 3).

**Figure 6:**
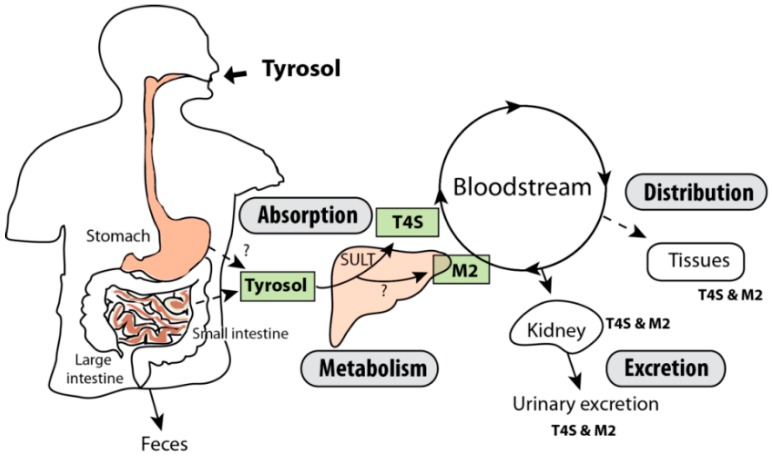
Proposed metabolic pathways of tyrosol after oral administration of tyrosol. T4S: tyrosol-4-sulfate; M2: tyrosol metabolite 2; SULT: sulfotransferase.

To study the tissue distribution of tyrosol metabolites, organs were collected at different time points following oral administration of tyrosol. T4S and M2 were detected in each tissue within 1 h after tyrosol administration (Figure 5A,B). The highest concentration of tyrosol-4-sulfate and M2 by tissue weight was observed in the liver and the kidney, while they were not detected in epididymal fat and the lungs. These findings indicate that the metabolites are mainly excreted by the kidneys through the liver. The proposed metabolic pathways of tyrosol in rats are shown in Figure 6. Specifically, we propose that the rapid uptake of tyrosol is followed by its sulfation in the liver.

The enzymes involved in phytochemical metabolism are present in many tissues, but, generally, these are more concentrated in the liver. Although metabolism typically inactivates the parent compound, some compounds are more potent as active metabolites [20]. A study suggests that tyrosol metabolites, tyrosol and hydroxytyrol sulfate, could protect from cell death induced by oxidized cholesterol and the biological effect of parent compounds can be retained in the metabolites [21]. One study reported that tyrosol treatment is more active than hydroxytyrosol for certain biological systems [22], although tyrosol has weak antioxidant activity compared with hydroxytyrosol *in vitro* [23]. The actual contribution of metabolites of parent compounds is still quite controversial. Our findings may suggest that T4S or M2 directly contributes to the health benefits of tyrosol. Indeed, tyrosol metabolites may exhibit biological activity in their target tissues.

## 3. Materials and Methods

### 3.1. Materials

Tyrosol was obtained from Sigma Chemical Co. (Deisenhofen, Germany). Tyrosol-4-sulfate was synthesized from Toronto Research Chemicals Inc. (Toronto, ON, Canada). HPLC-grade water, acetonitrile, and methanol were purchased from Fisher Scientific (Pittsburgh, PA, USA). All the other reagents were of analytical grade.

### 3.2. Treatment of Animals

Male Sprague-Dawley rats with average weights of 250 g were purchased from Orient Bio Inc. (Seoul, Korea) and acclimatized to laboratory conditions comprising a 12:12 h light-dark cycle, temperature of 24 °C, and humidity of 55% for one week. Animals were fed AIN-93G diet and provided water *ad libitum* during acclimation. On the dosing day, the rats were gavaged with tyrosol (100 or 200 mg/kg) as a suspension in polyethylene glycol. After the oral administration of a single dose of tyrosol to rats, blood samples were collected from the eye of the rats at different time points after dosing (0.25, 0.5, 1, 2, 4, 8, and 16 h). Urine samples were collected just before administration of tyrosol and during 0–1, 1–2, 2–4, 4–8, and 8–24 h after dosing. Liver, lung, heart, spleen, kidney, and epididymal adipose tissues were excised as quickly as possible and stored frozen at −80 °C until use. Animal studies were conducted in accordance with our institutional and national guidelines, and all experimental procedures were approved by the Korea Food Research Institute Animal Care and Use Committee (KFRI-IACUC, #2015-0016).

### 3.3. Plasma Extraction

To 300 µL of serum, 700 µL of methanol was added, followed by vortex mixing for 5 min. After centrifugation at 13,000 rpm for 15 min, the supernatant was collected and injected into the HPLC or LC-MS system.

### 3.4. Tissue Extraction

Each tissue was thawed, rinsed with ice-cold saline, blotted dry, weighed, and homogenized (1:1, *w*/*v*) in water using homogenizer (MP Fastprep instrument, MP Biomedical, Solon, OH, USA). The homogenates (300 µL) were transferred into clean tubes, methanol (700 µL) was added, and the tubes were vortex-mixed for 5 min. The supernatant was collected after centrifugation at 13,000 rpm for 15 min. The supernatant was evaporated to dryness at room temperature under a stream of nitrogen gas. The residue was resuspended in methanol (100 µL).

### 3.5. UPLC-MS Analysis

UPLC-MS was performed using an Acquity UPLC system (Waters, Miliford, MA, USA) coupled to an ultra-performance liquid chromatography-quadrupole time of flight mass spectrometry UPLC-ESI/Q-TOF (Waters Corp., Manchester, UK). An Acquity UPLC BEH C18 (2.1 × 100 mm, 1.7 μm) column was used at a column temperature of 30 °C and a flow rate of 0.30 mL/min. The mobile phase was composed of (A) 0.1% formic acid aqueous solution and (B) 0.1% formic acid in acetonitrile. Mobile phase conditions were as follows: initial condition of 98% A, 0–13 min with a gradient from 98%–5% A, 13–14 min at 5% A, and returning to 98% A for a 2 min re-equilibration step. The injection volume was 5 μL. A Waters Synapt G2-Si mass spectrometer was used and operated in electrospray ionization (ESI) mode. The ESI source was set in negative ESI mode with a scan range of *m*/*z* 50–1000. Argon was used as a collision gas, and nitrogen was used as a desolvation gas. The voltage of capillary, cone, and collision energy was set at 3.0 kV, 40 V, and 25 V, respectively. The desolvation and cone gas flow rates were 800 L/h and 50 L/h, respectively. The source temperature and desolvation gas temperature were 110 °C and 350 °C, respectively. All spectrum data were collected in continuum format using the MS^E^ acquisition mode. Mass accuracy was calibrated using sodium formate, and leucine enkephalin was used as lock mass. The concentration of leucine enkephalin was 2 ng/mL, and the flow rate was set at 5 µL/min. Raw data processing and instrument control were accomplished using the UNIFI software version 1.7.1. (Waters Inc.).

### 3.6. HPLC Analysis

HPLC analyses were performed on a Jasco high-performance liquid chromatography (HPLC) system (Jasco Corporation, Tokyo, Japan) equipped with a Jasco UV-2089 plus quaternary gradient pump, Jasco AS-2057 plus intelligent sample injector, Jasco UV-2075 UV/Vis detector, and Borwin chromatography software version 1.5. Separation was achieved using an XTerra RP18 column (4.6 × 250 mm, 5 µM, Waters) and a guard column. Elution was performed at a flow rate of 1.0 mL/min at 30 °C. The solvents for eluent were 1% (*v*/*v*) phosphoric acid (solvent A) and acetonitrile (solvent B). The solvent gradient changed according to the following conditions: 100% A to 95% A over 10 min, to 85% A over 10 min, to 70% A over 10 min, to 65% A over 5 min, to 90% A over 5 min, and to 100% A over 5 min, followed by 5 min of maintenance. The UV detector was set at a wavelength of 280 nm.

### 3.7. Analysis of Pharmacokinetics

A non-compartmental pharmacokinetics analysis for each tyrosol metabolite was performed using PK solution software version 2.0 (Summit Research Services, Montrose, CO, USA). C_max_, T_max_, and T_1/2_ were estimated from the plasma concentration-time curve. The area under the curve (AUC) for the plasma concentration over time was calculated using the trapezoidal rule.

## 4. Conclusions

The results of the present study indicate that after oral administration, tyrosol is absorbed rapidly and excreted via the kidney within 8 h. In particular, sulfation in the liver appears to be the major metabolic pathway of tyrosol, and its metabolites may exert various biological activities in tissues.

## Abbreviations

The following abbreviations are used in this manuscript:

T4S: Tyrosol-4-sulfate
UPLC-Q-TOF-MS: ultra-performance liquid chromatography and quadrupole time-of-flight mass spectrometry
HPLC: high-performance liquid chromatography
ROS: reactive oxygen species
C_max_: maximum plasma concentration
T_max_: time to maximum plasma concentration
T_1/2_: elimination half-life
